# Superstitions and practices of women in the perinatal period in Turkey: a cross-sectional study

**DOI:** 10.1590/1806-9282.20241206

**Published:** 2025-03-17

**Authors:** Fatma Şule Bilgiç, Aysu Yildiz Karaahmet

**Affiliations:** 1 Çanakkale Onsekiz Mart University, Faculty of Health Sciences, Department of Midwifery – Çanakkale, Turkey.; 2 Biruni University, Faculty of Health Sciences, Department of Midwifery – İstanbul, Turkey.

**Keywords:** Pregnancy, Maternity, Infant care, Traditional, Superstition

## Abstract

**OBJECTIVE:**

In this study, it was aimed to examine the superstitions and practices of women about pregnancy, birth, puerperium, and baby care.

**METHODS:**

The cross-sectional study was conducted on the online platform in May–June 2023 with 612 women across Turkey. The data were obtained through the "Data Collection Form" and the "Non-Functional Belief and Practices Scale."

**RESULTS:**

The study was completed with 612 women. Notably, 77.7% of the women participating in the study are single and 73.3% live in the city. Notably, 39.1% believed in traditional methods and 70.8% did not use a traditional practice. It was found that there was a significant difference between the total score of the scale and all its subdimensions, the place of living, and belief in traditional practices. The mean age of the women was 24.79±7.54 years, the NFBPS-P related to Pregnancy subdimension was 88.72±13.40, the NFBPS-CB related to Childbirth subdimension was 30.90±5.38, the NFBPS-P subdimension of Postpartum was 35.70±5.74, the NFBPS-IC related to Infant Care subdimension was 71.35±8.79, and the mean total score of the Non-Functional Belief and Practices Scale was 226.68±27.08. There was a positive correlation between the total score of the women and all subdimensions of the Non-Functional Belief and Practices Scale (p<0.05).

**CONCLUSION:**

It was found that women's nonfunctional beliefs and practices related to pregnancy, childbirth, puerperium, and baby care were high. It was determined that women living in the city and believing in traditional practices had higher beliefs and practices in nonfunctional practices.

## INTRODUCTION

The perinatal period is globally recognized as a key period for health intervention, encompassing pregnancy, childbirth, the postpartum period, and early infancy^1,2^. The concept of the "perinatal period" emerged in the late 1940s after clinicians and researchers became aware of high mortality rates before and after childbirth^3^. According to the World Health Organization, perinatal death includes stillbirths, that is, the death of a fetus that has completed the 21st week of gestation and the death of a newborn baby within 1 month of birth^4^. Almost all (98%) of the total number of perinatal deaths worldwide currently occur in developing countries^5^. Despite some progress in maternal and child health indices worldwide, inequalities remain, with the burden of illness and death emerging in resource-underfunded and unsustainable environments^6^.

Health care during the perinatal period, including pregnancy, childbirth, and postpartum care, is crucial not only to prevent neonatal deaths but also to effectively contribute to reducing stillbirth, as well as maternal mortality and disease^7,8^. Many international studies suggest that upward social determinants such as education levels, shared beliefs, values, practices, poverty, illiteracy, poor status, and women's care are important factors that negatively affect maternal and child health in many developing countries^8,9^. People's health-related beliefs and practices form part of the culture of the society in which they live. The inadequacy of the care provided in the postpartum period leads individuals to make traditional practices to solve their health problems. In addition, it is not necessary to go to any health institution unless it is very necessary for reasons such as the pressure of family elders, perceiving the problems seen in the postpartum period as a normal situation, the status of women in society, economic inadequacies, remoteness of health centers, religious beliefs, not trusting health personnel, and lack of health insurance. For these reasons, individuals try to solve their health problems with traditional practices they have seen from their families^6,8^. As seen in many studies on traditional practices in Turkey and in the world, although there are differences and similarities, it is seen that they are still being done^9,10^. Since 2010, more than 30 qualitative studies conducted in low-research environments have investigated the impact of cultures, values, and beliefs on the health of mothers and their babies^11,12^. While the results of the study show that traditional practices are used quite a lot in this period, no research has been conducted to fully understand the effects on mother and baby care in the perinatal period. In this study, it was aimed to examine the superstitions and practices of women about pregnancy, birth, puerperium, and baby care.

For this purpose, answers to the following questions were sought:

- What are the superstitions and practices that women apply during pregnancy, childbirth, and after childbirth?
- What are the superstitions and practices that women apply in baby care?

## METHODS

### Study design

The cross-sectional descriptive study aimed to examine women's nonfunctional beliefs and practices related to pregnancy, childbirth, puerperium, and infant care. During the investigation, the Strengthening the Reporting of Observational Studies in Epidemiology notification was monitored.

### Study population

The population of the study consists of all women in Istanbul, Turkey. For descriptive research, it was planned to reach 323 data, which is the maximum universe that can be taken with a margin of error of 0.05 and the minimum sample number^13^. In the data obtained through the snowball sampling method Google Forms, the link was left active for 10 days. The sample of the study consisted of 612 women who volunteered to participate in the study and met the sample selection criteria. All women aged 18 years and over, at least literate and able to use online links, were included in the study.

### Data collection tools

The data were collected using the Data Collection Form and the Non-Functional Belief and Practices Scale.

Data Collection Form: By reviewing the literature by researchers^8,9^, it consists of 10 questions on sociodemographic and traditional method belief.

Non-Functional Beliefs and Practices Scale (NFBPS): Yalçın and Koçak.^14^ The scale on which they conducted the Validity and Reliability Study consists of 58 items. It is desirable to indicate the extent to which the statements in the scale are appropriate for the volunteer. A statement is to be marked with the numbers "completely agree" (1), agree (2), undecided (3), disagree (4), and strongly disagree (5). Nonfunctional belief and practice covers practices that have been believed since time immemorial. A high score on the scale indicates that the woman has false information and beliefs about nonfunctional practices, while a low score on the scale indicates that her knowledge is healthier and more conscious. In total, the lowest score that can be taken from the scale is 58, which is the number of items, while the highest score is 290 points. The scale has four subdimensions. In this study, Cronbach's alpha reliability coefficient; "Non-Functional Beliefs and Practices of Pregnancy" 0.911; "Non-Functional Beliefs and Practices of Childbirth" 0.79; "Non-Functional Beliefs and Practices Regarding Puerperal" 0.804; and "Non-Functional Beliefs and Practices Regarding Infant Care" 0.799.

### Data collection process and bias

Survey access links were shared with women and information was given on how to fill out the questionnaire and send it online. To protect privacy, the survey is anonymous. There is also a statement at the beginning of the survey in which the women confirm in writing their willingness to participate in the study. The participation of women is free of charge, and it is informed that there is no benefit or harm.

### Analysis of data

The Statistical Package for Social Sciences (SPSS, 24.0) program was used to evaluate the findings of the study. Descriptive statistical analyses were obtained by frequency, percentage, mean (X), standard deviation, and min–max values. Whether the data were suitable for the normal distribution was evaluated by the Kolmogorov-Smirnov normality test. In the comparison of the Non-Functional Beliefs and Practices Scale scores and their descriptive characteristics, the Mann-Whitney U test was used for the evaluations between the two groups, and the Kruskal-Wallis test was used for the evaluations between more than two groups. The post hoc Bonferroni test was also used to determine the group that caused the difference. The results were evaluated using a 95% confidence interval, representing a significance level of 0.05 (p<0.05).

### Ethical approval

Within the scope of the study, ethical approval from an ethics committee (date: April 25, 2023; ethics committee no: 90) and the necessary permission was obtained from the author who developed the Non-Functional Belief and Practices Scale used in the research. Consent was obtained from the participants online before starting the research.

## RESULTS

The study was completed with 612 women. Notably, 77.7% of the women participating in the study are single and 73.3% live in the city. Notably, 39.1% believed in traditional methods and 70.8% did not use a traditional practice. It was also found that there was a significant difference between the total score of the scale and all its subdimensions, as well as the place of living and belief in traditional practices. According to the results of the analysis, it was found that there was a significant difference between women's place of residence and belief in traditional practices. It was determined that the mean of the total score of the place of residence and the NFBPS and its subdimensions was higher than the mean of the total score of the NFBPS and its subdimensions in the city and in the women living in the city and believing in traditional methods (Table 1; p<0.05).

**Table 1:** Distribution of categorical variables of women and comparison of Non-Functional Belief and Practices Scale and sub-dimensions (n=612).

Variables		n	%	NFBPS total	NFBPS-P	NFBPS-C	NFBPS-P	NFBPS-BC
Marital status	Married	137	22.3	219.65±24.78	85.38±12.545	29.40±5.321	34.86±5.32	70.00±7.44
	Single	477	77.7	228.70±27.39	89.67±13.50	31.33±5.32	35.94±5.84	71.74±9.11
	**U\p**			25,750\**0.000** *	25,611\**0.000** *	25,603\**0.000** *	28,259\**0.000** *	29,180\0.056
Where to live	Village (1)	26	4.2	227.07±24.68	88.00±14.43	30.69±4.78	36.84±5.01	71.53±6.74
	District (2)	138	22.5	218.68±29.74	85.44±15.14	29.55±5.82	34.60±5.94	69.08±8.44
	City(3)	450	73.3	229.12±25.91	89.76±12.62	31.33±5.21	35.97±5.69	72.04±8.90
	**X^2^ \p Bonferroni**			13.595\ ***0.001*** *** ***3>2***	8.695\**0.013** *** **3>2**	11.124\ ***0.004*** *** ***3>2***	6.185\ ***0.045*** *** ***3>2***	7.779\ ***0.020*** ** ***3>2***
Belief in traditional practices	Believes (1)	240	39.1	218.73±29.25	84.38±13.77	29.34±5.81	34.37±6.28	70.63±8.91
	Not believing (2)	176	28.7	239.29±23.57	94.99±10.84	33.17±4.32	37.43±4.85	73.71±9.25
	I'm undecided (3)	198	32.2	225.12±22.98	88.39±12.90	30.78±5.00	35.79±5.38	70.14±7.80
	**X^2^ /KW**			61.705\ ***000*** ** ***1>2***	66.970\ ***0.000*** ** ***1>2***	50.950\ ***0.000*** ** ***1>2***	25.25\ ***0.000*** ** ***1>3***	14.126\ ***0.001*** ** ***1>2***
Traditional practice	There is	179	29.2	218.82±28.93	84.76±14.10	29.60±5.84	34.06±6.06	70.38±8.42
	No	435	70.8	229.92±25.62	90.34±12.76	31.44±5.08	36.38±5.47	71.75±8.91
	U\p			29,669\ ***0.000*** *	29,421\*0.* ***000*** *	31,667\ ***0.000*** *	29,976\ ***0.000*** *	36,166\0.166*

NFBPS: Non-Functional Beliefs and Practices Scale; NFBPS-P: Non-Functional Beliefs and Practices Related to Pregnancy; NFBPS-C: Non-Functional Beliefs and Practices Regarding Chilbirth; NFBPS-P: Non-Functional Beliefs and Practices Regarding Puerperium; NFBPS-B: Non-Functional Beliefs and Practices Regarding Baby Care; SD: standart deviyasyon.

* Mann Whitney U;

** Kruskal-Wills;

*** Post Hoc Benferroni test p<0.05 anlamlı.

The average age of women is 24.79±7.54 years, NFBPS-P subdimension 88.72±13.40, NFBPS-CB subdimension 30.90±5.38, NFBPS-P subdimension 35.70±5.74, NFBPS-IC subdimension 71.35±8.79, and NFBPS* total average score 226.68±27.08. A positive relationship was found between women's NFBPS* total score and all subscales (p<0.05; Table 2).

**Table 2:** The relationship between the mean of women's continuous variables and Non-Functional Belief and Practices Scale and sub-dimensions (n=612).

Continuous variables	Mean±SD (minimum–maximum)	r/p	Age	NFBPS-P	NFBPS-CB	NFBPS-P	NFBPS-BC	NFBPS average total score
Age	24.79±7.54 (18.00-55.00)	**r**	1.000	-0.055	-0.046	-0.040	-0.077	-0.063
		**p**		0.172	0.258	0.322	0.058	0.120
NFBPS-P	88.72±13.40 (23.00-115.00)	**r**	-0.0055	1.000	0.778	0.580	0.264	0.882
		**p**	0.172		***0.000***	***0.000***	***0.000***	***0.000***
NFBPS-CB	30.90±5.38 (8.00-40.00)	**r**	-0.046	0.778	1.000	0.577	0.312	0.833
		**p**	0.258	***0.000***		***0.000***	***0.000***	***0.000***
NFBPS-P	35.70±5.74 (9.00-45.00)	**r**	-0.040	0.580	0.577	1.000	0.536	0.799
		**p**	0.322	***0.000***	***0.000***		***0.000***	***0.000***
NFBPS-BC	71.35±8.79 (18.00-90.00)	**r**	-0.077	0.264	0.312	0.536	1.000	0.601
		**p**	0.058	***0.000***	***0.000***	***0.000***		***0.000***
NFBPS average total score	226.68±27.08 (62.00-290.00)	**r**	-0.063	0.882	0.833	0.799	0.601	1.000
		**p**	0.120	***0.000***	***0.000***	***0.000***	***0.000***	

NFBPS: Non-Functional Beliefs and Practices Scale; NFBPS-P: Non-Functional Beliefs and Practices Related to Pregnancy; NFBPS-C: Non-Functional Beliefs and Practices Regarding Chilbirth; NFBPS-P: Non-Functional Beliefs and Practices Regarding Puerperium; NFBPS-B: Non-Functional Beliefs and Practices Regarding Baby Care; r: Sperm Correlation Test; p=signifidant value p<0.05 is significant.

## DISCUSSION

The study sheds light on the use of traditional medical practices practiced or believed in the perinatal period by women in Turkey. The study findings show that women use traditional methods in pregnancy, childbirth, and puerperal. In addition, the relationship between the women's ages, marital status, belief status in traditional practices, and the traditional practices they used and the NFBPS scale was significant.

Traditional practices are practiced by people not only because they are beliefs and traditions, but because they mean something to society and the individual. As seen in many studies on traditional practices in Turkey and in the world, although there are differences and similarities, it is seen that they are still being done^9,11^. In the study findings, it was found that women who believed in traditional practices had higher scores on the Non-Functional Beliefs and Practices Scale. A study in South Africa found that cultures, beliefs, and perceptions about pregnancy shape the traditional practices used during pregnancy. In the same study, it is believed that the fear of being attacked by evil spirits in women is greater than the fear of being affected by natural causes such as diabetes, asthma, or hypertension because supernatural evil spirits cause serious complications related to pregnancy, such as miscarriage and abnormal childbirth^11^. Likewise, other studies have indicated that the cultures and beliefs of communities influence and support a woman's traditional health-seeking behavior during pregnancy^15,16^. The study findings contradict evidence from Cambodia showing that harmful traditional practices that were once common in rural areas are no longer common and that the need to seek formal care for sick newborns, for example, is increasingly recognized^17^. These differences stem from a lack of understanding of the relationship between traditional cultural traditions and women's perceptions of perinatal care across developing countries, particularly in rural areas where access to quality prenatal and maternity care is limited.

This study showed that the sociodemographic characteristics of women such as age, place of residence, and marital status were used in traditional practices in the perinatal period. These findings reported that women living in the city were much more aware of the traditional methods used in the perinatal period than those living in the village. These findings are similar to those of a study conducted in Africa. In this study conducted in Africa, the employment levels of women did not affect their knowledge of the traditional medicinal products used in pregnancy by pregnant women^18^. Contrary to the findings of this study, other researchers have found that traditional intervention users are likely to be of lower socioeconomic and educational levels because they cannot afford access to health systems or pay for medical products^19^.

### Limitations and strengths

The fact that the study was conducted with a high sample is among its strengths. Because the scale used in the study is quite long, sociodemographic data were limited.

## CONCLUSION

The study findings show that traditional practices are still used in women. It requires supervising, evaluating, and strengthening the existing framework for integrating traditional health services used by women into the traditional health system. What the meaning of pregnancy, birth, and postpartum period in the society served, what are the cultural practices for these periods, and the identification of cultural barriers to health care in this period positively affect the care process. It is very important to know the traditional beliefs and practices used by societies, to reveal and try to eliminate those who are harmed by them, to protect those who are not harmful to maintain them, and to protect cultural features and traditions in a sense. Therefore, in order to provide better health services, it is necessary to understand how the cared-for group perceives disease and health and how they react to it without judging it.

## References

[1] Ahmed J, Alam A, Khokhar S, Khowaja S, Kumar R, Greenow CR (2020). Exploring women's experience of healthcare use during pregnancy and childbirth to understand factors contributing to perinatal deaths in Pakistan: a qualitative study. PLoS One.

[2] Ahmed J, Alam A, Rayenes-Greenow C (2018). Maternal empowerment and healthcare access determines stillbirths and early neonatal mortality in Pakistan: analysis of demographic and health survey 2012-13. J Glob Health Rep.

[3] Alderdice F (2017). The experience of stillbirth. J Reprod Infant Psychol.

[4] Alliance for Maternal and Newborn Health Improvement (AMANHI) mortality study group (2018). Population-based rates, timing, and causes of maternal deaths, stillbirths, and neonatal deaths in south Asia and sub-Saharan Africa: a multi-country prospective cohort study. Lancet Glob Health.

[5] Rowen T, Prata N, Passano P (2011). Evaluation of a traditional birth attendant training programme in Bangladesh. Midwifery.

[6] Aziato L, Odai PN, Omenyo CN (2016). Religious beliefs and practices in pregnancy and labour: an inductive qualitative study among post-partum women in Ghana. BMC Pregnancy Childbirth.

[7] Bhutta ZA, Das JK, Bahl R, Lawn JE, Salam RA, Paul VK (2014). Can available interventions end preventable deaths in mothers, newborn babies, and stillbirths, and at what cost?. Lancet.

[8] Bond D, Raynes-Greenow C, Gordon A (2018). Bereaved parents’ experience of care and follow-up after stillbirth in Sydney hospitals. Aust N Z J Obstet Gynaecol.

[9] Braun V, Clarke V (2006). Using thematic analysis in psychology. Qualitat Res Psychol.

[10] Christou A, Alam A, Hofiani SMS, Rasooly MH, Mubasher A, Rashidi MK (2019). How community and healthcare provider perceptions, practices and experiences influence reporting, disclosure and data collection on stillbirth: findings of a qualitative study in Afghanistan. Soc Sci Med.

[11] Francis JJ, Johnston M, Robertson C, Glidewell L, Entwistle V, Eccles MP (2010). What is an adequate sample size? Operationalising data saturation for theory-based interview studies. Psychol Health.

[12] Yazıcıoğlu Y, Erdoğan S (2004). SPSS Uygulamalı Bilimsel Araştırma Yöntemleri.

[13] Gedamu H, Tsegaw A, Debebe E (2018). The prevalence of traditional malpractice during pregnancy, child birth, and postnatal period among women of childbearing age in meshenti town, 2016. Int J Reprod Med.

[14] Yalçın H, Koçak N (2009). Fonksiyonel olmayan inanç ve uygulamalar tutum ölçeğinin geçerlik ve güvenirlik çalişmasi. Int J Soc Econ Sci.

[15] Zepro N (2015). Food taboos and misconceptions among pregnant women of shashemene district, ethiopia. Sci J Public Health.

[16] Otoo P, Habib H, Ankomah A (2015). Food prohibitions and other traditional practices in pregnancy: a qualitative study in western region of ghana. Adv Reprod Sci.

[17] Ayele G (2015). Prevalence and associated factors of home delivery in arbaminch zuria district, southern ethiopia: community based cross sectional study. Sci J Public Health.

[18] Zenebe K, Alem H, Merga A, Abate G, Taha H (2015). Prevalence of cultural malpractice and associated factors among women attending MCH clinic at debretabor governmental health institutions south gondar, amhara region, north west Ethiopia. Gyneco Obstet.

[19] Legesse M, Demena M, Mesfin F, Haile D (2015). Factors associated with colostrum avoidance among mothers of children aged less than 24 months in Raya Kobo district, North-eastern Ethiopia: community-based cross-sectional study. J Trop Pediatr.

